# Screening of Neutrophil Activating Factors from a Metagenome Library of Sponge-Associated Bacteria

**DOI:** 10.3390/md19080427

**Published:** 2021-07-28

**Authors:** Yoshiko Okamura, Hirokazu Takahashi, Atsuyuki Shiida, Yuto Hirata, Haruko Takeyama, Katsuhiko Suzuki

**Affiliations:** 1 Graduate School of Integrated Sciences for Life, Hiroshima University, Higashihiroshima 739-8530, Japan; ziphiro@hiroshima-u.ac.jp (H.T.); m210634@hiroshima-u.ac.jp (Y.H.); 2 Graduate School of Advanced Sciences of Matter, Hiroshima University, Higashihiroshima 739-8530, Japan; m206282@hiroshima-u.ac.jp; 3 Department of Life Science and Medical Bio-Science, Waseda University, Tokyo 162-8480, Japan; haruko-takeyama@waseda.jp; 4 Faculty of Sport Sciences, Waseda University, Saitama 359-1192, Japan

**Keywords:** activity-based screening, bioactive compound, metagenome analysis, neutrophil activating peptide

## Abstract

Marine sponge-associated bacteria are known as bio-active compound produce. We have constructed metagenome libraries of the bacteria and developed a metagenomic screening approach. Activity-based screening successfully identified novel genes and novel enzymes; however, the efficiency was only in 1 out of 10^4^ clones. Therefore, in this study, we thought that bioinformatics could help to reduce screening efforts, and combined activity-based screening with database search. Neutrophils play an important role for the immune system to recognize excreted bacterial by-products as chemotactic factors and are recruited to infection sites to kill pathogens via phagocytosis. These excreted by-products are considered critical triggers that engage the immune system to mount a defense against infection, and identifying these factors may guide developments in medicine and diagnostics. We focused on genes encoding amino acid ligase and peptide synthetase and selected from an in-house sponge metagenome database. Cell-free culture medium of each was used in a neutrophil chemiluminescence assay in luminol reaction. The clone showing maximum activity had a genomic sequence expected to produce a molecule like a phospho-*N*-acetylmuramyl pentapeptide by the metagenome fragment analysis.

## 1. Introduction

The immune system plays an important role in the prevention of bacterial infections. Neutrophils undergo a chemotaxis, which allows them to migrate toward sites of infection or inflammation. Neutrophils recognize chemotactic factors (chemoattractants) via cell surface receptors [1]. Detection of chemotactic factors by their receptors triggers the activation of cellular movement and results in quickly congregating at an infection site by attraction by cytokines expressed by activated endothelium, mast cells, and macrophages or by bacterial by-products [2]. In humans, neutrophil chemoattractants belong to four biochemically distinct subfamilies, i.e., chemokine (C-X-C motif) ligand (CXCL) family (CXCL1 to CXCL3 and CXCL5 to CXCL8 in humans), complement anaphylatoxins (C3a and C5a), chemotactic lipids (e.g., leukotriene B4 or LTB4), and formyl peptides [3]. A well-known example of a formyl peptide is *N*-formyl-Met-Leu-Pro (fMLP), which was synthesized by mimicking bacteria. *N*-formylmethionine is commonly used for an initial amino acid of bacterial protein; therefore, Schiffman et al. demonstrated *N*-formylmethioninyl peptides showing chemoattractant activity as for resembling chemotactic factors produced by bacteria [4]. In addition to fMLP, there are no other peptide ligands reported for a long time, and the discovery of new ligands is a very important research target in the field of drug discovery.

Peptides are generally generated by cleaving proteins or by enzymatic ligation of amino acids. The enzymatic method employs amino acid ligases, which are ATP-dependent enzymes [5]. The enzymatic method is more cost-effective and leads to milder conditions than the solid-phase synthesis method; however, owing to substrate specificity, the variety of peptides is limited by a single kind of amino acid ligase. Therefore, it is necessary to prepare many kinds of amino acid ligases for the enzymatic method for industrial production.

Amino acid ligases have been discovered mainly from microorganisms. The discovery of new amino acid ligases has been performed by homology search using the sequences of known enzymes or by selecting candidate sequences that have unique ATP-binding domains in the ligase, and finally succeeded in expressing them in host cells such as *Escherichia coli* for functional analysis [6]. As this approach requires the full-length sequence information of the genes or genomic sequence, it is mainly performed on established strains. However, standard culturing techniques support the establishment of less than 1% of the bacteria found in the environment [7]. More than 99% of the microorganisms in natural environment are reported to be difficult or impossible to culture, so-called ‘visible but non-culturable’ (VBNC) bacteria [8]. Therefore, the VBNC bacteria must be ignored and not to be used for a source of novel enzyme screening and a potential source of functional genes. For this reason, metagenome analyses have gained popularity for the screening and identification of genes of interest. It is expected that, with the metagenome library, it would be possible not only to use the genetic information of VBNCs, but also to express them in the host cells such as *E. coli* and determine the activity to meet the non- elucidated characteristics or undetermined phenotypic properties. In fact, we have reported a novel esterase [9] and a novel gene involved in cadmium accumulation [10] from sponge-associated bacterial metagenome by library screening. We have constructed two types of sponge-associated bacterial metagenome libraries derived from *Hyrtios erecta* [9] and *Stylissa massa* [10] because marine sponges are known to be one of the largest producers of sondary metabolites [11] and hold high potential of harboring unique functional genes including those related to heavy metal accumulation [12,13].

On the other hand, we have used 26,496 clones of a plasmid library [9] and 3301 clones of a fosmid library [10] for a functional screening approach, so it is not easy work to cultivate a large number of clones. To solve this problem of inefficiency, we have determined DNA sequences of about 2 kbp at both ends in every single vector from the metagenome library and an established in-house database to combine in silico screening with functional screening. All DNA sequences were analyzed and annotated based on homology and domain, and registered in the in-house database.

In this study, at first, we selected 120 candidates from the in-house database for amino acid ligases and peptide synthases using clusters of orthologous groups (COG) analysis or selected by Pfam analysis of the ATPases associated with diverse cellular activities (AAA) domain, an ATP-binding motif that is a conserved domain involved in peptide synthethase, and then the gene cluster activating neutrophils was identified.

## 2. Results

### 2.1. Selection of Positive Clones

**Figure 1:**
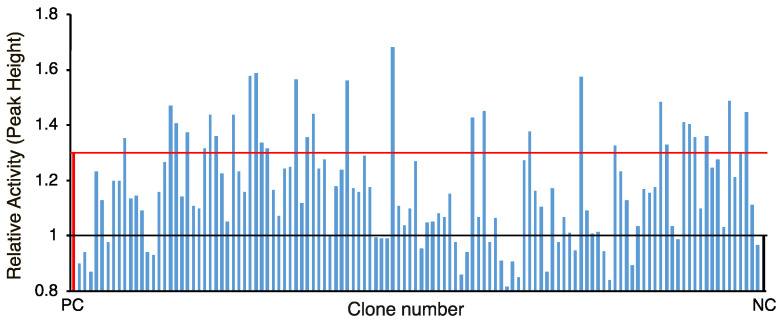
Neutrophil stimulating activities of selected 120 clones. The activity is shown as relative activity to *E. coli* harboring empty vector (negative control; NC). *N*-formyl-Met-Leu-Pro (fMLP) (2 ng) was used as positive control (PC). Black and red lines indicate relative activities of negative control and positive control, respectively.

**Figure 2:**
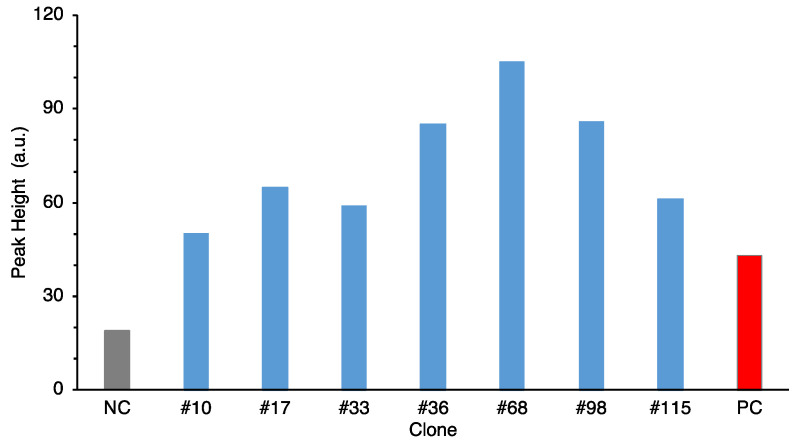
Neutrophil stimulating activities of further selected clones at third screening. The activity is shown as maximum luminol chemiluminescent intensity (a.u.). NC: HBSS buffer, PC: 2 ng of fMLP. Grey bar: NC (negative control), red bar: PC (positive control).

As mentioned above, candidates were selected based on COG and pfam and 120 clones (shown in Supplementary Table S1) were examined for second screening. Neutrophil assay employed boiled culture supernatant to detect chemiluminescent intensity [14]. Commercial chemotactic factor fMLP (Sigma-Aldrich, St. Luis, MO, USA) was used as positive control and supernatant form culture of *E. coli* harboring empty vector (pHSG398) was used as negative control. As shown in Figure 1, there were 18 clones showing greater peak height than 1.3 times the value of the negative control. The positive control (2 ng of fMLP) was 1.3. The assay was repeated five times or more and 7 out of 120 clones were selected with reproducibility for the next candidates (Figure 2).

**Figure 3:**
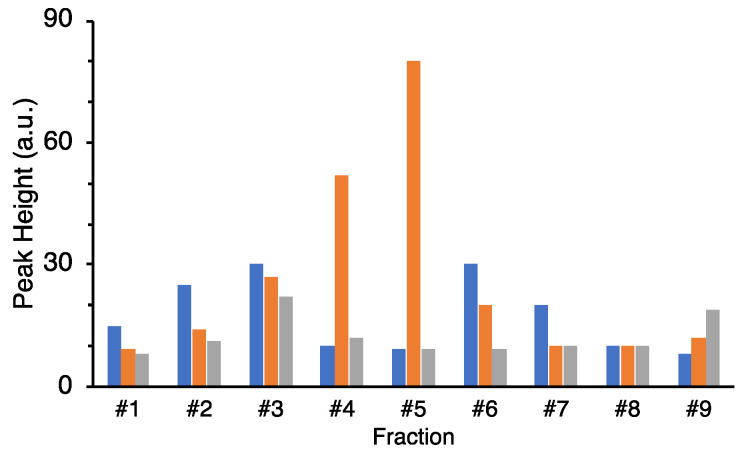
Fractionation of culture supernatant including secreted factors from clone #98 and #115 and neutrophil stimulating activities of each fraction. Blue bar: #98, orange bar: #115, gray bar: *E. coli* (negative control).

**Figure 4:**
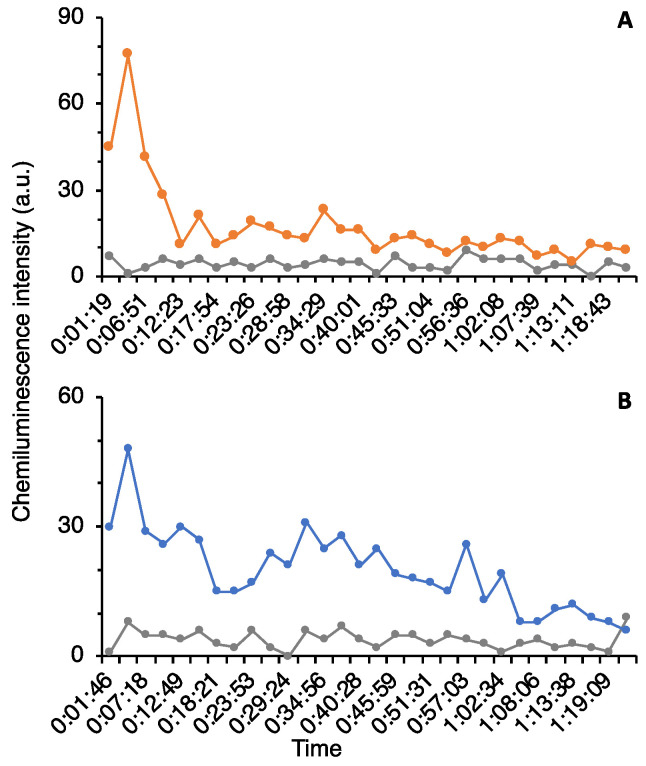
Time course of luminol chemiluminescent intensities in the fraction #5 from clone #115 (**A**) and fraction #6 from clone #98 (**B**). Gray color showed the activity of same fraction from *E. coli* (negative control).

Next, the culture supernatants of these seven clones were purified by HPLC to identify the fractions containing neutrophil stimulating activity. However, the fractions from 5 out of 7 candidates did not show significant activities after fractionation by HPLC. As the results, clone #98 and #115 contained significant activities compared with the correspondent fraction from the negative control (Figure 3). The fractions from clone #98 showed less activity than the fractions from clone #115. According to Figure 3, fraction #5 from clone #115 and fraction #6 from clone #98 were chosen and determined time course changes of luminescence intensity (Figure 4). The chemoattractants found in clone #115 and #98 were characterized by an immediate activation of neutrophils followed by a rapid decrease in response. The peak height of fraction #5 in clone #115 was the greatest; therefore, we chose it for further purification. The activity of fraction #6 from clone #98 was unstable, so it was not applied to further experiment.

### 2.2. Purification of Chemoattractant

**Figure 5:**
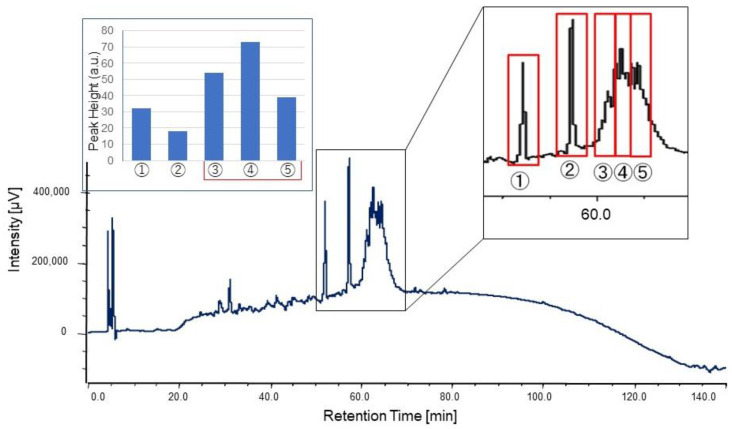
Further fractionation of culture supernatant from clone #115 and positive fractions (magnified chromatogram) and their activity (inserted bar graph).

Fraction #5 in clone #115 was collected up to 25 mL and lyophilized. The concentrated fraction was prepared by being redissolved in 20 µL of milliQ and applied on a C18 column. Elution employed a linear gradient of 0–100% acetonitrile (solvent A: 0.l% TFA/water; solvent B: 0.l% TFA/acetonitrile) for 120 min at a flow rate of 0.4 mL/min. Figure 5 shows the chromatogram and every peak was collected and assayed. As a result of the assay, the main body of activity was distributed into three peaks between 60 and 62 min. Each of these three fractions was subjected to Orbi-Trap MS analysis; however, the fractions still contained several substances and it was difficult to estimate the de novo sequence of the peptide. As it was hard to identify the peptide sequence, we decided to identify the responsible genes of the peptide synthesis.

### 2.3. Sequence Analysis of Metagenomic Fragment of Clone #115

The nucleotide sequence of the metagenomic fragment of strain #115 originated from *Hyrtios erecta*-associated bacteria was determined by primer walking and the sequence of 2992 bp was obtained. The fragment contained 3 ORFs; however, one promoter region was only found before the last ORF. Therefore, it was considered that the *lac* promoter of the vector would be used to express former ORFs (Supplementary Figure S1A). Moreover, under the *lac* promoter, ribosome binding site and start codon derived from the vector region were fused to the upstream of metagenomic fragment and another ORF was formed by in-frame fusion. Thus, four ORFs inferred inside the metagenomic fragment, and ORF1 to 3, were assumed to form an operon. Deduced amino acid sequences of each ORF were analyzed by BLASTP [15] and the homology is shown in Table 1. ORF1 and ORF3 showed highly homology with UDP-*N*-acetylmuramoylalanyl-d-glutamate-2,6-diaminopimelate ligase; however, ORF1 lacked one out of three domains consisting of the homologous protein. It has been reported that, among three domains, the central domain has an ATP-binding function and the *C*-terminal domain has ATP- and substrate-binding functions [16]. Therefore, it was assumed that ORF1 might work for the operon.

ORF4 is presumed to be a phospho-*N*-acetylmuramoyl-pentapeptide-transferase, suggesting that it modifies the products produced by ORF1–3. However, ORF4 was truncated so that the conserved domain was not completed (Supplementary Figure S1B). Phospho-*N*-acetylmuramoyl-pentapeptide-transferase has 10 transmembrane regions [17], but ORF4 lacked the sequence after the seventh transmembrane region.

### 2.4. Construction of Recombinant Strains Expressing Combined Several ORFs

**Figure 6:**
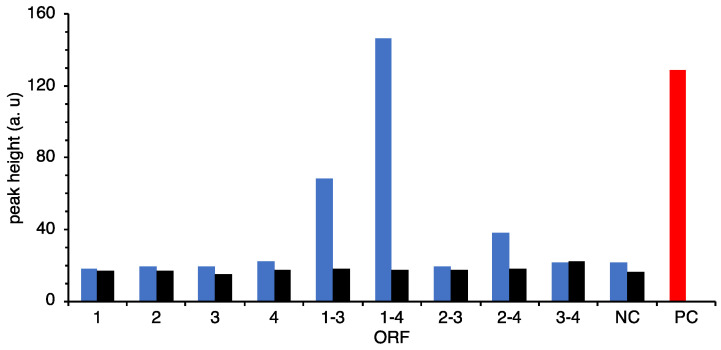
Identification of coding genes of neutrophil stimulating activity using the overexpression system. Protein expression was induced by lactose. *E. coli* harboring empty vector (NC) was also induced by lactose. Blue bar: induction, black bar: non-induction, red bar: 5 ng of fMLP for positive control.

Recombinant strains harboring the expression vector shown in Supplementary Figure S2 were prepared and neutrophil stimulation activities were assayed. The assay results showed that the recombinant retaining all of ORF1–4 had the highest values (Figure 6). ORF1–3 retained strains also had relatively high values, but it was about half of ORF1–4 retained strains. ORF2–4 retained strains also showed small activity. This result suggests that ORF4 was involved in the increase in activity and ORF2 was required for the function of ORF4. This may be due to the fact that ORF4 modifies the products of ORF1–3, but considering the activity of ORF3–4, it is highly likely that ORF4 modifies the products of ORF2–3.

## 3. Discussion

Although the *N*-terminal domain of ORF1 and *C*-terminal domain of ORF4 were deleted, the remaining domains of both ORFs are still functional and the expected peptide was obtained. However, it is not possible to affirm that it is the natural product of the ORF1–4 because of the missing domains. Moreover, ORF1 is required for showing activity suggesting that both products of ORF1 and ORF3 would be combined to form pentapeptide and modified by a phospho-*N*-acetylmuramoyl-pentapeptide-transferase of ORF4. ORF2 did not show any significant similarity; however, it would be required for ORF4 (Figure 6). The genes of ORF1, 3, and 4 showed homologies with the genes involved in peptidoglycan biosynthesis in *E. coli* cells [18]. Peptidoglycan consists of linear glycan chains interlinked by short peptides. The glycan chains are composed of alternating units of *N*-acetylglucosamine and *N*-acetylmuramic acid. Muramyl residues bear short pentapeptides. Peptidoglycan is synthesized by the key enzymes MurA to MurF. The *mur*A to *mur*F genes are all essential in bacteria. MurE, ATP-dependent amino acid ligase, converts UDP-*N*-acetylmuramyl-l-alanine-d-Glutamate to UDP-*N*-acetylmuramyl-tripeptide with meso-diaminopimelic acid. MurF catalyses the addition of d-alanyl d-alanine dipeptide to UDP-*N*-acetylmuramyl-tripeptide with meso-diaminopimelic acid and forms UDP-*N*-acetylmuramyl-pentapeptide [18]. ORF1 would retain the part of MurE activity. ORF4 showed homology with MraY, which converts UDP-*N*-acetylmuramyl-pentapeptide with undecaprenyl-phosphate into undecaprenyl-diphosphate-*N*-acetylmuramyl-pentapeptide and UMP [19].

**Figure 7:**
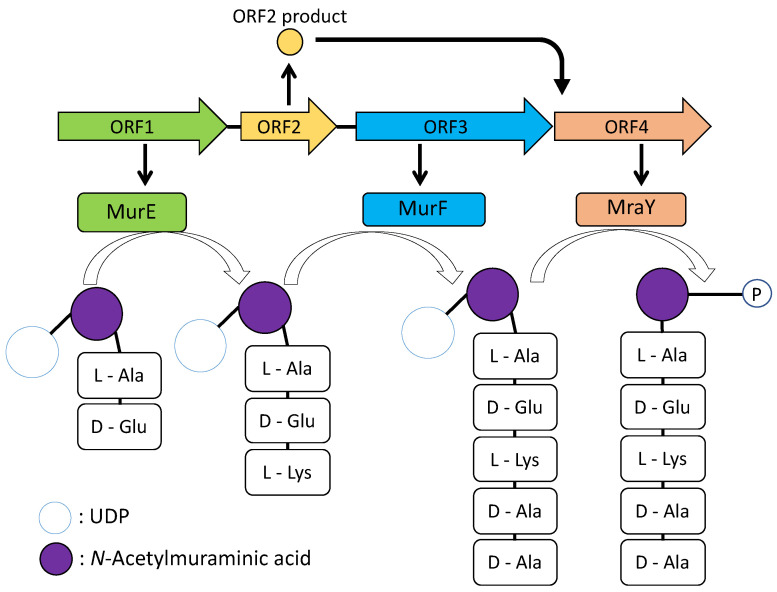
Hypothesized product of the gene cluster encoded in the metagenome fragment.

Based on the peptideglycan biosynthetic pathway, the function of the metagenomic fragment was hypothesized (Figure 7). As MurE and MurF are homologous to ORF1 and ORF3, respectively, the sequence of the peptide chain might be similar to l-Ala-d-Gln-l-Lys-d-Ala-d-Ala or different. As MraY is homologous to ORF4, it may function to add an undecaprenyl-diphosphate to *N*-acetylmuramyl-pentapeptide; however, ORF4 is also not in a complete form, and this pentapeptide might be secreted from the cells to the medium, not bound to the cell wall. In addition, as *mur*A, *mur*B, *mur*C, and *mur*D genes are necessary for peptidoglycan synthesis, they were not included in the metagenomic fragment, and the glycol-pentapeptide would be in an incomplete form. It could be considered that native genes from host *E. coli* complemented the functions. A result from de novo sequence through Orbi-trap MS, however, did not correspond to accurate mass of Ala and Lys, indicating that peptide sequence would be different from l-Ala-d-Gln-l-Lys-d-Ala-d-Ala. According to Tabata et al. [5], amino acid ligase is known for low substrate specificity and accepts a wide variety of l-amino acids. We considered the reason that MS fragments were shown to be complicated and did not show known accurate mass might be that several sequences were contained. Based on the gene analysis, the neurophil activating peptide, at least, would be predicted as the pentapeptide modified by *N*-acetylmuramic acid.

Along with improving next generation sequence, metagenome determination became easier and less costly, and could be utilized for gene screening based on the conserved sequence or homology search. As a result, the identified genes show similar characteristics to known genes and the chance to meet novel genes and enzymes is less than the functional screening. Because of the limitation, we have conducted activity-based screening for metagenome analysis so far. The construction of a metagenome library and activity-based screening spent much time and effort; however, to express them in the host cells of *E. coli* and determine the activity can lead to meeting the non-elucidated characteristics or undetermined phenotypic properties. Moreover, the in-house database for metagenome libraries was also established, thus we were able to compare the screening efficiency between general activity-based screening and activity-based screening combined with in silico screening, because of the same libraries. As mentioned above, we used 26,496 clones to identify a novel esterase gene [9]. Similarly, a xylanase gene was selected among 40,000 clones from the soil metagenome library [20]. Thus, we estimated the efficiency of activity-based screening was 1 out of 10^4^ clones. In this study, we cultivated only 120 clones by *in silico* screening and the efforts were reduced by 1/100 and the efficiency increased to 100-fold. In addition to this approach’s merit, we could access the candidates and propagate the recombinants once the libraries were constructed.

## 4. Materials and Methods

### 4.1. Metagenomic Libraries

Metagenomic libraries used in this study have been established in Okamura et al. [9] and Mori et al. [10]. LB medium containing chloramphenicol (Cm; final concentration: 20 μg/mL) was used for bacterial culture.

### 4.2. In Silico Screening

For the first screening, amino acid ligase or peptide synthase genes were selected from our in-house database. Specifically, the genes contain an adenylation domain, which is required for activation by ATP to form a peptide bond. COG search and Pfam domain searches were employed to select ligase and AAA domains. The clones that conserved full-length ORFs were selected.

### 4.3. Sample Preparation for Neutrophil Activity Assay

For the second screening, all 120 selected clones were grown in 96-DeepWell plate (maximum volume 2 mL/well) (Nunc^TM^, Nalge Nunc International, New York, NY, USA) with 1 mL of LB (Cm) with continual agitation at 170 rpm in a Bio-Shaker (TITEC, Saitama, Japan) at 37 °C for 8 h. After centrifugation at 10,000× *g* for 5 min, supernatant was boiled at 80 °C for 20 min.

For the third screening, selected clones were cultured in L-shaped test tube (id: 18 mm, custom-made in Hiroshima University) with 5 mL of LB (Cm) with horizontal shaking at 120 rpm at 37 °C for 8 h. After centrifugation and boiling, the same as above, the supernatant was lyophilized, redissolved in milliQ, and concentrated at fivefold.

### 4.4. Purification

The culture supernatant after boiling was lyophilized, redissolved in milliQ, concentrated 5–10 times, and used for HPLC purification. The concentrated supernatant was applied on a C18 column (TSKgel ODS-80Ts, Tosoh, Yamaguchi, Japan) and eluted with a linear gradient of 0–100% acetonitrile (solvent A: 0.l% TFA/water; solvent B: 0.l% TFA/acetonitrile) in 8–40 min for 32 min at a flow rate of 1.0 mL/min. After linear gradient, mobile phases at 100% and 0% acetonitrile were kept for 5 min, respectively. A column oven was used to keep the column at 40 °C during the measurement. The eluate was monitored at 230 nm with a MD-2010 multiwavelength photodiode array detector (JASCO Cooperation, Tokyo, Japan). Fractions were obtained at the first 10 min (Fraction #1) and at intervals of 5 min/fraction (Fraction #2–9). For further purification, the same mobile phase was used at a flow rate of 0.4 mL/min in 120 min with linear gradient. The fractions were separated by peak appearance. The obtained fractions were completely dried by decompression drying and re-dissolved in 20 μL of Hanks’ Balanced Salt Solution (HBSS (1×): GIBCO^®^, Thermo Fisher Scientific, Waltham, MA, USA) for the assay.

### 4.5. Neutrophil Activating Assay

The assay using neutrophils was based on the method of Hasegawa et al. [14]. Whole blood was employed in the assay. The principle is based on the NADPH oxidase activity from neutrophils, which receive the chemoattractants, and the resulting superoxide anion reacts with luminol, thus the activity can be detected as a luminescent intensity. Luminol solution was prepared as follows: 17.72 mg of luminol (SIGMA) was dissolved in 3 mL of 1N NaOH, then 2.5 mL of 1N HCl was added. Then, 30 mL of HBSS (-) (HBSS (1×) without Ca^2+^ and Mg^2+^) was added, adjusting pH to pH = 7.4 (37 °C) with 0.2 N HCl, and finally mess up to 40 mL with HBSS (1×). For the assay, the luminol solution was diluted five times with HBSS (1×) and used. The blood samples were donated by male and female volunteers in their 20s to 40s. To prevent blood coagulation, 10 μL of 10 U/μL heparin (Wako Pure Chemical Corporation, Tokyo, Japan) dissolved in 50 μL of HBSS (1×) was added to the Falcon in advance. Blood was kept at 37 °C as much as possible. A 96-well plate was used for measurement. To all wells, 20 μL of boiling supernatant or purified fraction and 60 μL of reaction mixture of blood and luminol solution in equal volume were added, and the measurement was started immediately. The plate reader was set up so that there was a 10 s shaking step immediately before scanning, and the luminescence in each well was measured for 30 cycles of 3 min each, including a 170 s waiting period after scanning, for an overall measurement time of 90 min. The fluorescence plate reader was ARVO (Perkin Elmer, Waltham, MA, USA) or Fluoroskan Ascent FL (ThermoFisher Scientific, Waltham, MA, USA). Nunclon^®^ 96 well (bottom clear, flat bottom, black, product no. 165305) (ThermoFisher Scientific, Waltham, MA, USA) was used for measurement. The highest value in the assay was used as the peak height for comparison.

### 4.6. Nucleotide Sequence Analysis of Metagenomic Insertion Fragments

The plasmid DNA was extracted from positive clones showing neutrophil activation, and the sequence of the metagenome fragment was sent for sequencing by Takara Bio Inc. (Shiga, Japan). ORFs were predicted by a free software ApE (A plasmid editor, v2.0.61) (https://jorgensen.biology.utah.edu/wayned/ape/, accessed on 10 February 2020). The sequences were analyzed by Basic Local Alignment Search Tool (BLAST) [15]. Promoter regions were predicted using GENETYX Ver.9 (GENETYX CORPORATION, Tokyo, Japan).

### 4.7. Construction of Expression Vectors and Recombinants

In order to determine the responsible gene(s) for neutrophil activation, four ORFs from the metagenome fragment were cloned separately or several clusters into pGEX-6p-1 expression vector. PCR primers to amplify each ORF or gene cluster were listed in Supplemental Table S2. Restriction enzyme sites within the multi-cloning site were employed, and translation was initiated at the vector start codon. KOD-Plus-Neo (TOYOBO, Osaka, Japan) was used with 2 µL of the extracted plasmid (10 ng/μL) as template and 1.5 μL of each primer (10 pmol/μL) in 50 µL of total volume. The thermal cycling conditions were as follows: pre-denaturation at 94 °C for 2 min, followed by 30 cycles of denaturation at 98 °C for 10 s, annealing and extension at 68 °C for 30 s for amplicon length <500 bp, 45 s for <800 bp, 60 s for <1 kbp, 75 s for <1.3 kbp, 105 s for <1.7 kbp, or 150 s for 2.3 kbp. The resulting PCR product was ligated into the appropriate sites downstream of the promoter in the pGEX-6p-1 vector and cloned in *E. coli* DH5a and plated on LB agar medium supplemented with ampicillin at 50 μg/mL. Colony PCR was performed to check for insertion of specific open reading frames (ORFs) in the recombinant clones. KOD Dash (TOYOBO) was used for colony PCR. Then, 0.5 μL of each primer (10 pmol/μL) was used for the reaction, and the total volume was 20 μL. The used primers were pGEX 5’ (5’-GGGCTGGCAAGCCACGTTTGGTG-3’) and pGEX 3’ (5’-CCGGGAGCTGCATGTGTCAGAGG-3’). The thermal cycling conditions were as follows: pre-denaturation at 95 °C for 4 min, followed by 35 cycles of denaturation at 98 °C for 20 s, annealing at 60 °C for 10 s, and extension at 72 °C for 2 min, with a final extension at 60 °C for 4 min. The PCR products were confirmed by electrophoresis in 1% agarose gel. The resulting transformants were grown in LB medium supplemented with ampicillin (50 µg/mL) and culture supernatant of each transformant was collected to be used for assay.

### 4.8. Accession Number

The sequence of metagenomic fragment was deposited to DDBJ (LC634461).

## 5. Conclusions

Activity-based metagenome library screening combined with in silico screening increased the screening efficiency from 1 out of 100,000 clones to 1 out 120 clones. The positive clone showing neutrophil stimulating activity harbored incomplete peptideglycan biosynthesis genes. The product of the metagenome fragment was expected to be the phospho-*N*-acetylmuramyl pentapeptide; however, the peptide was not able to be purified and identified.

## Figures and Tables

**Table 1:** BLASTP sequence similarity search using the NCBI non-redundant protein database.

ORF	Homology	Score	Identity (%)	E-Value	Coverage	Organisms	Accession#
ORF1 (267aa)	UDP-*N*-acetylmuramoyl-l-alanyl-d-glutamate-2,6-diaminopimelate ligase	411 bits(1056)	91	1 × 10^−139^	245/473 aa	*Rhodospirillales bacterium*	MYE18720.1
ORF2 (151aa)	No significant similarity found	-	-	-	-	*-*	-
ORF3 (311aa)	UDP-*N*-acetylmuramoyl-tripeptide-d-alanyl-d-alanine ligase	550 bits(1416)	94	0.0	310/458 aa	*Rhodospirillales bacterium*	MXX22569.1
ORF4 (249aa)	phospho-*N*-acetylmuramoyl-pentapeptide-transferase	448 bits(1153)	98	3 × 10^−156^	231/362 aa	*Rhodospirillales bacterium*	MXX22568.1

## Data Availability

No new data were created or analyzed in this study. Data sharing is not applicable to this article.

## Supplementary Materials

The following are available online at https://www.mdpi.com/article/10.3390/md19080427/s1, Figure S1: Arrangement of the open reading frames in the metagenomic fragment of clone #115 (**A**) and the truncated regions of homologous proteins in ORF1 and ORF4 (**B**); Figure S2: Expression vector constructs for single ORF expression or co-expression. The inserts were amplified by PCR and cloned into MCS of oGEX-6p-1; Table S1: List of primers designed for gene expression; Table S2: List of selected clones by in silico screening.
