# Supplementary material for: Screening of Neutrophil Activating Factors from a Metagenome Library of Sponge-Associated Bacteria
Source: Mar Drugs. 2021 Jul 28;19(8):427. doi: 10.3390/md19080427 (PMC8402132; doi:10.3390/md19080427)
Supplement: Supplementary file 1 [file marinedrugs-19-00427-s001.zip › Proof for Supplemental data.pdf]

Screening of Neutrophil Activating Factors from a Metagenome Library of Sponge-Associated Bacteria (TENTATIVE)

Yoshiko Okamura, Hirokazu Takahashi, Atsuyuki Shiida, Yuto Hirata, Haruko Takeyama, Katsuhiko Suzuki

Supplemental Figures and Tables

Supplemental Table S1.   List of selected clones by *in silico* screening

Clone #	Register #	Originated library	annotation
1	201-B10	Styllisa massa Fosmid	Ubiquitin-protein ligase
2	204-H07	Styllisa massa Fosmid	D-alanine-D-alanine ligase and related ATP-grasp enzymes
3	96-E02	Hyrtios erecta plasmid	D-alanine-D-alanine ligase and related ATP-grasp enzymes
4	314-A11	Styllisa massa Fosmid	Ubiquitin-protein ligase
5	165-G12	Styllisa massa Fosmid	PPE-repeat proteins; Predicted ATPase (AAA+ superfamily)
6	167-H02	Styllisa massa Fosmid	Predicted ATPase (AAA+ superfamily)
7	168-F05	Styllisa massa Fosmid	D-alanine-D-alanine ligase and related ATP-grasp enzymes
8	101-F08	Styllisa massa Fosmid	UDP-N-acetylmuramyl pentapeptide synthase
9	101-A09	Styllisa massa Fosmid	UDP-N-acetylmuramyl pentapeptide synthase
10	224-H06	Styllisa massa Fosmid	UDP-N-acetylmuramyl pentapeptide synthase
11	97-D01	Styllisa massa Fosmid	MoxR-like ATPases; AAA ATPase containing von Willebrand factor type A (vWA) domain
12	97-G03	Styllisa massa Fosmid	Phosphoribosylaminoimidazole (AIR) synthetase
13	372-F11	Styllisa massa Fosmid	Folate-dependent phosphoribosylglycinamide formyltransferase PurN
14	40-F03	Hyrtios erecta plasmid	Non-ribosomal peptide synthetase modules and related proteins
15	62-E09	Hyrtios erecta plasmid	UDP-N-acetylmuramyl pentapeptide synthase
16	63-E06	Hyrtios erecta plasmid	Folylpolyglutamate synthase
17	65-F04	Hyrtios erecta plasmid	UDP-N-acetylmuramoylalanine-D-glutamate ligase
18	66-D10	Hyrtios erecta plasmid	D-alanine-D-alanine ligase and related ATP-grasp enzymes
19	67-B03	Hyrtios erecta plasmid	UDP-N-acetylmuramoylalanine-D-glutamate ligase
20	24-H07	Hyrtios erecta plasmid	Folylpolyglutamate synthase
21	27-G11	Hyrtios erecta plasmid	UDP-N-acetylmuramyl pentapeptide synthase
22	28-F03	Hyrtios erecta plasmid	Predicted unusual protein kinase
23	30-B01	Hyrtios erecta plasmid	UDP-N-acetylmuramoylalanine-D-glutamate ligase
24	31-F12	Hyrtios erecta plasmid	UDP-N-acetylmuramate-alanine ligase
25	201-F06	Hyrtios erecta plasmid	Non-ribosomal peptide synthetase modules and related proteins
26	22-D07	Hyrtios erecta plasmid	Non-ribosomal peptide synthetase modules and related proteins
27	23-D12	Hyrtios erecta plasmid	UDP-N-acetylmuramoylalanine-D-glutamate ligase
28	81-G09	Hyrtios erecta plasmid	UDP-N-acetylmuramate-alanine ligase
29	85-B10	Hyrtios erecta plasmid	Non-ribosomal peptide synthetase modules and related proteins
30	85-D06	Hyrtios erecta plasmid	UDP-N-acetylmuramyl tripeptide synthase; UDP-N-acetylmuramoylalanine-D-glutamate ligase
31	89-B02	Hyrtios erecta plasmid	Non-ribosomal peptide synthetase modules and related proteins
32	91-C11	Hyrtios erecta plasmid	Formyltetrahydrofolate synthetase
33	93-D03	Hyrtios erecta plasmid	D-alanine-D-alanine ligase and related ATP-grasp enzymes
34	179-F03	Styllisa massa Fosmid	D-alanine-D-alanine ligase and related ATP-grasp enzymes
35	181-F01	Styllisa massa Fosmid	Folylpolyglutamate synthase
36	181-G02	Styllisa massa Fosmid	Folate-dependent phosphoribosylglycinamide formyltransferase PurN
37	189-B09	Hyrtios erecta plasmid	UDP-N-acetylmuramate-alanine ligase
38	192-E03	Hyrtios erecta plasmid	UDP-N-acetylmuramyl tripeptide synthase
39	193-C12	Hyrtios erecta plasmid	Non-ribosomal peptide synthetase modules and related proteins
40	204-G09	Hyrtios erecta plasmid	Non-ribosomal peptide synthetase modules and related proteins
41	206-H01	Hyrtios erecta plasmid	Folylpolyglutamate synthase
42	210-D04	Hyrtios erecta plasmid	Formyltetrahydrofolate synthetase
43	210-D11	Hyrtios erecta plasmid	Folylpolyglutamate synthase
44	212-G09	Hyrtios erecta plasmid	Folylpolyglutamate synthase
45	213-D03	Hyrtios erecta plasmid	UDP-N-acetylmuramyl tripeptide synthase; UDP-N-acetylmuramyl pentapeptide synthase
46	214-H01	Hyrtios erecta plasmid	Folylpolyglutamate synthase
47	2-B09	Hyrtios erecta plasmid	Formyltetrahydrofolate synthetase
48	4-C03	Hyrtios erecta plasmid	UDP-N-acetylmuramyl pentapeptide synthase
49	4-D10	Hyrtios erecta plasmid	UDP-N-acetylmuramoylalanine-D-glutamate ligase
50	6-H02	Hyrtios erecta plasmid	D-alanine-D-alanine ligase and related ATP-grasp enzymes
51	12-C03	Hyrtios erecta plasmid	Formyltetrahydrofolate synthetase
52	13-A04	Hyrtios erecta plasmid	D-alanine-D-alanine ligase and related ATP-grasp enzymes
53	33-C05	Hyrtios erecta plasmid	Non-ribosomal peptide synthetase modules and related proteins
54	38-F05	Hyrtios erecta plasmid	Folate-dependent phosphoribosylglycinamide formyltransferase PurN
55	52-C04	Hyrtios erecta plasmid	UDP-N-acetylmuramyl pentapeptide synthase
56	54-D09	Hyrtios erecta plasmid	Folylpolyglutamate synthase
57	54-F05	Hyrtios erecta plasmid	UDP-N-acetylmuramyl tripeptide synthase
58	58-C04	Hyrtios erecta plasmid	Formyltetrahydrofolate synthetase
59	68-B06	Hyrtios erecta plasmid	Folate-dependent phosphoribosylglycinamide formyltransferase PurN
60	72-E08	Hyrtios erecta plasmid	D-alanine-D-alanine ligase and related ATP-grasp enzymes
61	73-B12	Hyrtios erecta plasmid	D-alanine-D-alanine ligase and related ATP-grasp enzymes
62	73-H09	Hyrtios erecta plasmid	UDP-N-acetylmuramoylalanine-D-glutamate ligase
63	78-C05	Hyrtios erecta plasmid	UDP-N-acetylmuramyl tripeptide synthase
64	96-D08	Hyrtios erecta plasmid	D-alanine-D-alanine ligase and related ATP-grasp enzymes
65	96-E02	Hyrtios erecta plasmid	D-alanine-D-alanine ligase and related ATP-grasp enzymes
66	97-B04	Hyrtios erecta plasmid	Non-ribosomal peptide synthetase modules and related proteins; Peptide arylation enzymes
67	100-G12	Hyrtios erecta plasmid	5-formyltetrahydrofolate cyclo-ligase
68	139-F08	Hyrtios erecta plasmid	D-alanine-D-alanine ligase and related ATP-grasp enzymes
69	141-C04	Hyrtios erecta plasmid	UDP-N-acetylmuramoylalanine-D-glutamate ligase
70	143-C01	Hyrtios erecta plasmid	Non-ribosomal peptide synthetase modules and related proteins
71	143-G03	Hyrtios erecta plasmid	UDP-N-acetylmuramyl pentapeptide synthase; UDP-N-acetylmuramyl tripeptide synthase
72	186-F06	Hyrtios erecta plasmid	Folate-dependent phosphoribosylglycinamide formyltransferase PurN
73	103-C09	Hyrtios erecta plasmid	D-alanine-D-alanine ligase and related ATP-grasp enzymes
74	104-D10	Hyrtios erecta plasmid	Folate-dependent phosphoribosylglycinamide formyltransferase PurN
75	104-F11	Hyrtios erecta plasmid	UDP-N-acetylmuramyl tripeptide synthase
76	105-F10	Hyrtios erecta plasmid	Formyltetrahydrofolate synthetase
77	112-A08	Hyrtios erecta plasmid	UDP-N-acetylmuramoylalanine-D-glutamate ligase
78	114-C03	Hyrtios erecta plasmid	UDP-N-acetylmuramoylalanine-D-glutamate ligase
79	114-C10	Hyrtios erecta plasmid	UDP-N-acetylmuramyl pentapeptide synthase
80	120-F09	Hyrtios erecta plasmid	D-alanine-D-alanine ligase and related ATP-grasp enzymes
81	121-F11	Hyrtios erecta plasmid	Folylpolyglutamate synthase
82	123-H09	Hyrtios erecta plasmid	Non-ribosomal peptide synthetase modules and related proteins;
83	125-D02	Hyrtios erecta plasmid	Formyltetrahydrofolate synthetase
84	129-A07	Hyrtios erecta plasmid	UDP-N-acetylmuramyl tripeptide synthase
85	137-B09	Hyrtios erecta plasmid	UDP-N-acetylmuramyl tripeptide synthase; UDP-N-acetylmuramyl pentapeptide synthase
86	140-B04	Hyrtios erecta plasmid	D-alanine-D-alanine ligase and related ATP-grasp enzymes
87	18-C04	Hyrtios erecta plasmid	D-alanine-D-alanine ligase and related ATP-grasp enzymes
88	146-C03	Hyrtios erecta plasmid	UDP-N-acetylmuramyl pentapeptide synthase
89	147-E02	Hyrtios erecta plasmid	UDP-N-acetylmuramyl pentapeptide synthase
90	147-F11	Hyrtios erecta plasmid	UDP-N-acetylmuramoylalanine-D-glutamate ligase
91	150-F01	Hyrtios erecta plasmid	UDP-N-acetylmuramyl tripeptide synthase
92	152-E10	Hyrtios erecta plasmid	UDP-N-acetylmuramyl tripeptide synthase
93	161-B04	Hyrtios erecta plasmid	UDP-N-acetylmuramate-alanine ligase
94	164-H10	Hyrtios erecta plasmid	D-alanine-D-alanine ligase and related ATP-grasp enzymes
95	165-H12	Hyrtios erecta plasmid	Folylpolyglutamate synthase
96	169-H12	Hyrtios erecta plasmid	UDP-N-acetylmuramoylalanine-D-glutamate ligase
97	174-A03	Hyrtios erecta plasmid	Folylpolyglutamate synthase
98	183-A02	Hyrtios erecta plasmid	Non-ribosomal peptide synthetase modules and related proteins;
99	178-A01	Hyrtios erecta plasmid	Predicted ATPase (AAA+ superfamily)
100	175-G11	Hyrtios erecta plasmid	D-alanine-D-alanine ligase and related ATP-grasp enzymes
101	181-H05	Hyrtios erecta plasmid	D-alanine-D-alanine ligase and related ATP-grasp enzymes
102	184-F03	Hyrtios erecta plasmid	D-alanine-D-alanine ligase and related ATP-grasp enzymes
103	180-A04	Hyrtios erecta plasmid	FOG: Ankyrin repeat
104	180-B10	Hyrtios erecta plasmid	Non-ribosomal peptide synthetase modules and related proteins;
105	171-F08	Hyrtios erecta plasmid	Formyltetrahydrofolate synthetase
106	22-F10	Hyrtios erecta plasmid	D-alanine-D-alanine ligase and related ATP-grasp enzymes
107	20-D11	Hyrtios erecta plasmid	UDP-N-acetylmuramyl tripeptide synthase
108	19-A04	Hyrtios erecta plasmid	Formyltetrahydrofolate synthetase
109	45-H06	Hyrtios erecta plasmid	Predicted unusual protein kinase
110	53-F11	Hyrtios erecta plasmid	UDP-N-acetylmuramyl tripeptide synthase
111	61-G06	Hyrtios erecta plasmid	Folate-dependent phosphoribosylglycinamide formyltransferase PurN
112	59-G11	Hyrtios erecta plasmid	Folate-dependent phosphoribosylglycinamide formyltransferase PurN
113	57-H05	Hyrtios erecta plasmid	UDP-N-acetylmuramate-alanine ligase
114	05-H04	Hyrtios erecta plasmid	D-alanine-D-alanine ligase and related ATP-grasp enzymes
115	32-F04	Hyrtios erecta plasmid	UDP-N-acetylmuramyl tripeptide synthase
116	34-D08	Hyrtios erecta plasmid	Non-ribosomal peptide synthetase modules and related proteins
117	39-H09	Hyrtios erecta plasmid	UDP-N-acetylmuramyl pentapeptide synthase
118	40-D11	Hyrtios erecta plasmid	UDP-N-acetylmuramate-alanine ligase
119	41-B06	Hyrtios erecta plasmid	UDP-N-acetylmuramyl tripeptide synthase; UDP-N-acetylmuramyl pentapeptide synthase
120	42-H04	Hyrtios erecta plasmid	Formyltetrahydrofolate synthetase

Supplemental Table S2. List of primers designed for gene expression

No.	Name	sequence
1	EcoRI_ORF1_-2F	GAG AGA GAA TTC TCA GTG CTC GAG GCC TCG TCC CAT GGC
2	EcoRI_ORF2_1F	GAG AGA GAA TTC ATG CAC TCC TGG TTG CCG GCA AGG GGC
3	EcoRI_ORF3_1F	GAG AGA GAA TTC ATG CCG GCC GCC ACT TCA GCG GCC GTA TTC
4	EcoRI_ORF4_1F	GAG AGA GAA TTC ATG CTG TTC CAT TTC CTG GTT CCG CTC
5	NotI_ORF1_754R	GAG AGA GCG GCC GCT CAG GCG CTC CCC GCA GCA CCG GCA CG
5	SbfI(PstI)_ORF1_754R	GAG AGA CCT GCA GGT CAG GCG CTC CCC GCA GCA CCG GCA CG
6	XhoI_ORF2_456R	GAG AGA CTC GAG TCA GCT CCT TGG TGC CGG TCT TGC CGA C
7	NotI_ORF3_936R	GAG AGA GCG GCC GCT CAG TGC GCC TTC GAC CCG GTG ACC CG
7	SbfI(PstI)_ORF3_936R	GAG AGA CCT GCA GGT CAG TGC GCC TTC GAC CCG GTG ACC CG
8	NotI-PstI_ORF4_693R	GAG AGA GCG GCC GCC TGC AGA TAG TCC GCA AAG AAC GCG TTG
2	EcoRI_ORF2_pHSG398	GAG AGA GAA TTC AAT GCA CTC CTG GTT GCC GGC AAG GGG C
3	EcoRI_ORF3_pHSG398	GAG AGA GAA TTC AAT GCC GGC CGC CAC TTC AGC GGC CGT ATT C
4	EcoRI_ORF4_pHSG398	GAG AGA GAA TTC AAT GCT GTT CCA TTT CCT GGT TCC GCT C
9	M13-P8	AGC GGA TAA CAA TTT CAC ACA GGA AAC
10	M13-P7	CGC CAG GGT TTT CCC AGT CAC GAC

The restriction enzyme recognition sequence was indicated by underline.

M13-P8 and M13-P7 are primers derived from the vector sequence, and the restriction enzyme digestion site used is the sequence in the multi-cloning site of the vector.

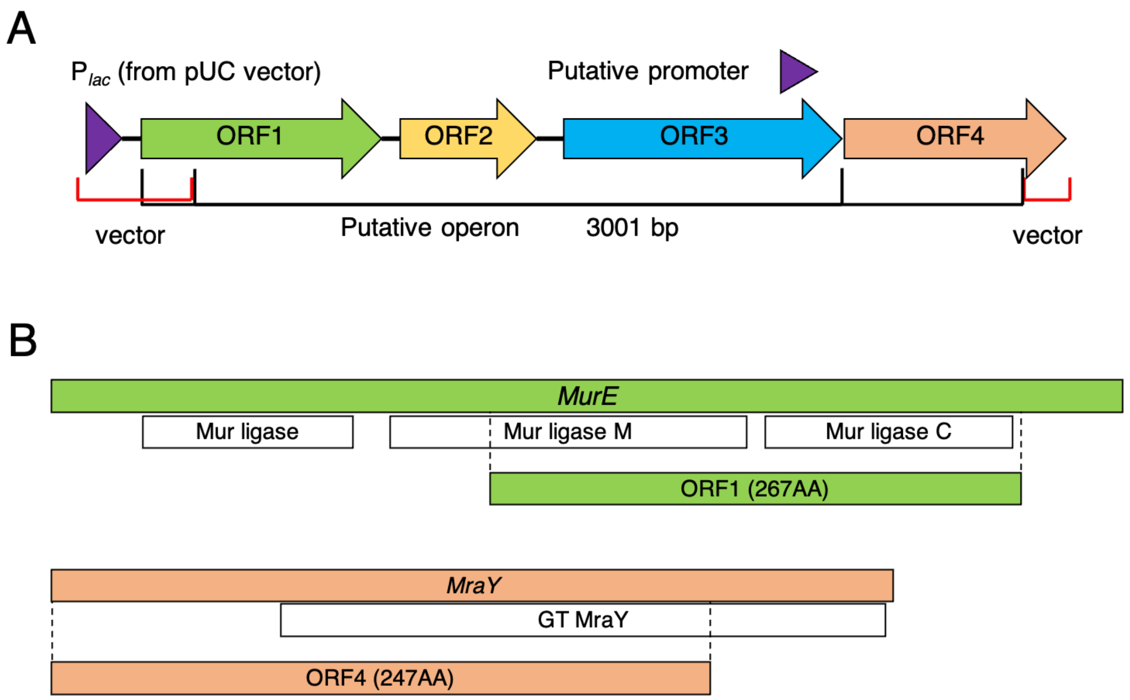

Figure S1. Arrangement of the open reading frames in the metagenomic frangment of clone #115 (A) and the truncated regions of homologous proteins in ORF1 and ORF4 (B).

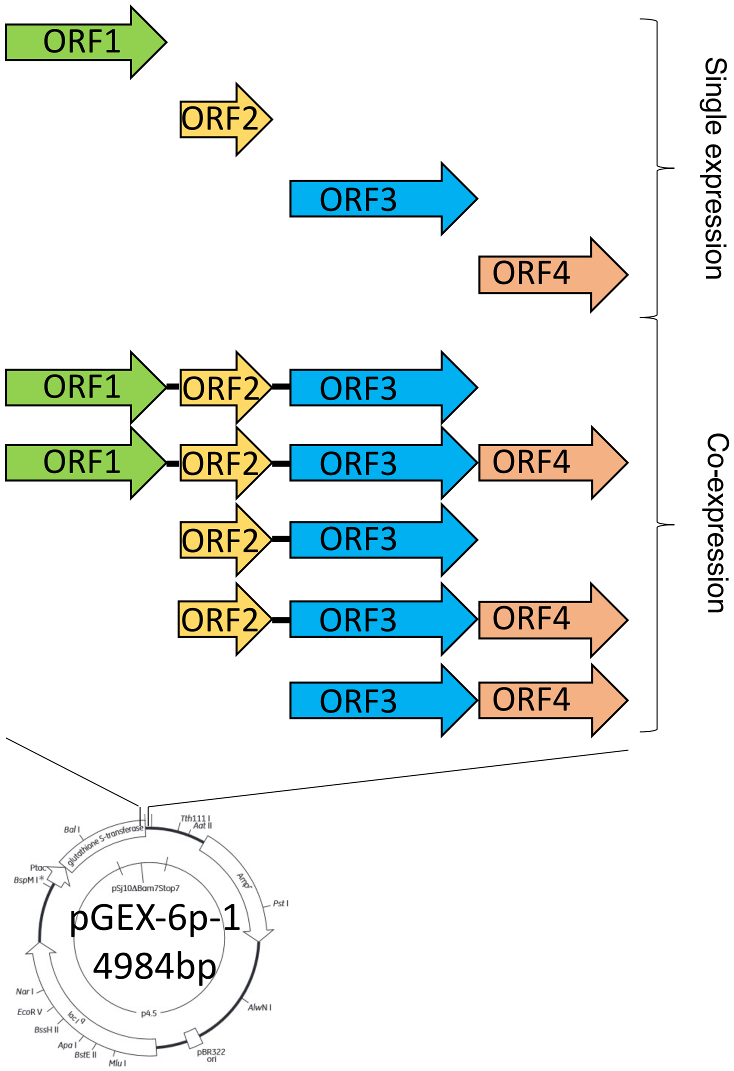

Figure S2. Expression vector constructs for single ORF expression or co-expression. The inserts were amplified by PCR and cloned into MCS of oGEX-6p-1.
